# Silicon in the Soil–Plant Continuum: Intricate Feedback Mechanisms within Ecosystems

**DOI:** 10.3390/plants10040652

**Published:** 2021-03-30

**Authors:** Ofir Katz, Daniel Puppe, Danuta Kaczorek, Nagabovanalli B. Prakash, Jörg Schaller

**Affiliations:** 1 Dead Sea and Arava Science Center, Mt. Masada, Tamar Regional Council, 86910 Tamar, Israel; 2 Eilat Campus, Ben-Gurion University of the Negev, Hatmarim Blv, 8855630 Eilat, Israel; 3 Leibniz Centre for Agricultural Landscape Research (ZALF), 15374 Müncheberg, Germany; daniel.puppe@zalf.de (D.P.); Danuta.Kaczorek@zalf.de (D.K.); Joerg.Schaller@zalf.de (J.S.); 4 Department of Soil Environment Sciences, Warsaw University of Life Sciences (SGGW), 02776 Warsaw, Poland; 5 Department of Soil Science and Agricultural Chemistry, University of Agricultural Sciences, GKVK, Bangalore 560065, India; nagabovanalliprakash@rediffmail.com

**Keywords:** silicon, soil, plants, cycling, ecosystem, services, feedback

## Abstract

Plants’ ability to take up silicon from the soil, accumulate it within their tissues and then reincorporate it into the soil through litter creates an intricate network of feedback mechanisms in ecosystems. Here, we provide a concise review of silicon’s roles in soil chemistry and physics and in plant physiology and ecology, focusing on the processes that form these feedback mechanisms. Through this review and analysis, we demonstrate how this feedback network drives ecosystem processes and affects ecosystem functioning. Consequently, we show that Si uptake and accumulation by plants is involved in several ecosystem services like soil appropriation, biomass supply, and carbon sequestration. Considering the demand for food of an increasing global population and the challenges of climate change, a detailed understanding of the underlying processes of these ecosystem services is of prime importance. Silicon and its role in ecosystem functioning and services thus should be the main focus of future research.

## 1. Introduction

Silicon (Si) uptake and accumulation is a functional trait with multiple implications for plant biology and ecology [1,2]. Silicon’s manifold functions in plant biology include protection from a myriad of abiotic and biotic stresses and confers many benefits to plants that are capable of taking up and accumulating large amounts of it, ranging from practically zero in some taxa to 5% dry weight (and in extreme cases even more) in grasses [1,3,4,5,6,7,8,9,10,11,12,13,14,15,16,17], and probably even to those plants that take up considerably smaller amounts [5,18]. Moreover, its uptake from the soil and eventual reincorporation into the soil through plant litter and herbivore feces also affects soil properties and Si cycling [19,20,21,22,23]. Thus, Si uptake, accumulation and cycling by plants is a key phenomenon in many ecosystems [12,21,24,25,26], with direct and indirect implications for ecosystem properties and processes [1]. Here, we review the existing knowledge of Si in the soil–plant continuum, its roles in plant biology and ecology, in ecosystem processes, and the possible implications for various ecosystem services.

## 2. Historical Overview

In 1787, Antoine Lavoisier predicted the existence of the element Si, to which Sir Humphry Davy proposed the name “silicon” in 1808. It was eventually isolated and formally discovered in 1823 by Jöns Jacob Berzelius. The discovery of the occurrence of Si within plants quickly followed, owing to the works of some prominent scholars, including microscopy pioneer Christian Gottfried Ehrenberg (who suggested the word “phytolitharia” to describe plant mineral components) [27] and Charles Darwin, who provided him with some samples [28,29]. Silicon effects on plant performance are known for more than 170 years, starting with Struve in 1835 [30], and shortly after Davy’s pioneering publication from 1846, who suggested that Si is present in the epidermis of grasses, where it strengthens the plants and makes them more resistant to attacks by insects and “parasitical plants” [31]. A surge of research soon followed. Sachs [32] showed in 1862 that Si-accumulating plants were a less preferred food than their conspecific plants that were grown hydroponically in Si-poor media. He further found that Si accumulation started in plant hairs and further advanced into the epidermis and near leaf vascular tissues. He also suggested that not all Si deposits in plant leaves are hard, but some may remain in a colloidal state. Miliarakis [33] found in 1884 that basal (younger) leaf parts of Si-accumulating plants had lower Si concentrations than older leaf tips and could not deter feeding. He also found that horsetail (*Equisetum*) and sedge (*Carex* and *Scirpus*) old leaf sheaths had a high Si concentration and protected the younger and less silicified plant tissues from herbivory. Furthermore, in 1884, Kreuzhage and Wolff [34] suggested the importance of Si for oat plants. Kerner von Marilaun [35] suggested in 1887 that the sharp leaf margin of sedges may be due to Si deposits. In 1888, Stahl [36] summarized other studies and concluded that silica deposits in horsetails impeded grazing by snails. He also mentioned that plant tissue silicification reduced the food quality of tropical grasses in Africa for domestic animals.

A second surge of studies started in the 1920s. Between 1922 and 1925, Lemmermann and colleagues [37,38,39] found an increase in the yield of rye grown under phosphorus deficiency upon Si fertilization. Sommer [40] concluded in the year 1926 that rice plants without Si fertilization suffered from early increased leaf wilting, guttation, and retarded panicle formation. In parallel, a surge of studies on Si effects on rice took place in Japan, starting in the 1910s and continuing into the 1940s, summarized thoroughly by Ma and Takahashi [41]. In the 1910s, Onodera [42] found that blast-infected rice leaves had a lower Si concentration than healthy leaves. Miyake et al. [43] also found in 1922 that Si concentration was higher in blast-resistant plants than nonresistant (surprisingly, this specific Japanese research was published in German). Other studies showed an increased blast-resistance of rice plants following Si application [44,45]. In the 1930s Ishibashi [46,47,48] showed reduced growth and yield of rice plants under Si deficiency and reduced blast after Si fertilization. Raleigh [49] showed in 1939 that Si was strongly improving the growth of beet plants. A year later, Wagner [50] showed that Si protects plants against powdery mildew by silicifying host plants’ cell walls, hence reducing penetration.

The study of silicon in plants continued shortly following the end of World War II, and especially since the 1960s. Engel [51] found in 1953 that the Si accumulated in rye culm was ~1/3 easily (hot water) extractable, suggesting no strong binding of this fraction to the plant cell walls. Holzapfel and Engel [52] found that Si accumulation in wheat increases over a study period of 30 days. In 1962, Yoshida [53] discovered the cuticle–Si double layer and suggested that this layer may be responsible for plant resistance against fungal diseases. There were several publications by Okuda and Takahashi, who found that Si improves plant resistance to metals [54] and rice growth and nutrition of [55]. Many of these studies were reviewed by Lewin and Reimann at the end of the 1960s [56]. During the same period, Jones and his colleagues (mainly Handreck) published some seminal papers on the occurrence, uptake, localization and functions of Si in oats [57,58,59,60] and clover [61]. At around the same time, a group that developed around Parry and his successor Sangster (both of which have passed away only in the past decade in their 90 s) further looked into the physiology of Si uptake and deposition [62,63,64,65,66,67,68,69,70,71]. Their early studies set the basis for a greater effort in Si plant and soil research, and a third surge that started in the 1980s and continues to these days, initiated by many important scientists and continuing to these days by the many who follow their footsteps.

## 3. Soluble and Particulate Silicon in the Soil

This section provides a short overview of the different forms of Si in soils and the main drivers for Si availability for plants. A more elaborate review of Si occurrence forms, speciation and cycling in soils is provided by Schaller et al. [26] in another manuscript published within this Special Issue.

Silicon occurs in many forms in the soil. The solid forms of soil Si include crystalline forms of primary (e.g., quartz, feldspars, micas) and secondary silicate minerals (e.g., the different clay minerals), as well as microcrystalline or poorly crystalline minerals (e.g., allophane, imogolite, opal-CT) [72,73]. Amorphous silica (ASi) includes both Si of mineralogical origins that is included in pedogenic oxides (e.g., iron oxides) and biogenic Si (e.g., phytoliths) [72,73]. All these can undergo dissolution, under various conditions and rates, to become the source of dissolved plant-available monosilicic and polysilicic acids (H_4_SiO_4,_ jointly termed “silicic acid” hereafter) [72] with a maximum solubility of ~1 mM. Both acids, in turn, may adsorb to soil particles (e.g., Fe or Al oxides/hydroxides) [72,74,75]. Polysilicic acid is mobilized during the dissolution of Si-rich solids [76], whereas at equilibrium Si in solution occurs as monosilicic acid [77]. However, at the early stages of dissolution, the presence of polymeric species may account for approximately 50% (by mol) of total dissolved silica [76]. Polysilicic acid converts over time to monosilicic acid if the concentration is far below saturation, but with increasing silicic acid concentrations in solution, the polymerization of monosilicic acid to polysilicic acid occurs. Nevertheless, silicic acid in soil solution is not only available for plant uptake and can precipitate but may also bind to (secondary) minerals [75]. The absorption of polysilicic acid to mineral surfaces is much faster than the sorption of monosilicic acid and is reversible; therefore, monosilicic acid is more abundant in soil porewaters than polysilicic acid [78]. In a study of soils from Karnataka, southern India, ASi was found to be the most abundant soil Si fraction, whereas silicic acid and adsorbed Si were the least abundant [79], and that high soil ASi contents are often associated with high contents of the clay size fraction [79,80], both a consequence of long-term primary silicate dissolution and plant activity.

### 3.1. Silicic Acid Effects on Nutrient and Toxicant Availability in Soils

Although silicic acid is competing with nutrients for binding to soil mineral surfaces [81,82,83], Si fertilization has been suggested to increase plant P status by potentially increasing P availability [84,85]. A recent study showed that increasing Si availability in soils leads to a mobilization of Fe(II)-P phases from mineral surfaces, thus increasing P availability/mobility in soils [83]. Silicic acid can be assumed to be a non-charged bidentate ligand at soil pH < 7 [86]. Thus, pH has a strong influence on soil Si availability [79,87]. The monosilicic acid molecule is in direct competition for sorption sites with the monodentate and bidentate (depending on pH) ligands of the phosphate molecule [88]. Despite having weaker binding energy to iron oxides than phosphate [86], silicic acid may outcompete phosphate if its concentration in pore water is sufficiently high. Another important property of silicic acid is that it can polymerize at high concentrations both in solution and on iron oxide surfaces forming Si–O–Si oligomer chains [89,90]. The binding affinity of polysilicic acid seems to be higher than monosilicic acid [76]. This may explain why silicic acid at higher concentrations is able to outcompete phosphate at binding sites of soil minerals. A further effect of Si in soils is the occupation of soil binding sites by an excess of silicic acid preventing nutrient binding to soil mineral surfaces [88].

Not only nutrients like P and N are mobilized from soils by Si [81,91], but also potentially toxic metals and metalloids (e.g., Al, Cr, Cd, Pb and Zn) [81] that impair plant performance if taken up [92]. However, less is known about how silicic acid potentially mobilizes metals and metalloids from different soil minerals. It is known that high pH Si fertilizers (such as fly ash or steel slag) decrease metal availability due to changing metal speciation and/or increased precipitation [93]. The bioavailability of aluminum may be reduced by Si fertilization owing to instant binding of Al to Si [26,94,95] to form hydroxyl aluminosilicates [96]. Such a co-precipitation with Si was also suggested for Cr, Cd, Pb and Zn [97,98,99]. However, it was shown that Cr immobilization by binding to iron minerals was reduced after pretreatment of the iron minerals with silicic acid [100]. Hence, in systems with high Si availability, ASi precipitations on mineral surfaces potentially act as a long-term hindrance for immobilization of toxic metals and metalloids as the binding of those toxic elements to mineral surfaces may be blocked [88].

### 3.2. Amorphous Silica as Control for Water Availability in Soils

Some literature claimed that ASi increases soil water holding capacity [73,88,101], possibly via silica gel formation from polysilicic acid or colloidal ASi [102]. A recent study showed that ASi strongly increases soil water holding capacity and availability for plants [103]. An increase of 1% or 5% ASi (by weight) increased the water content at any water potential and plant-available water by up to 40% or 60%, respectively [103]. However, a comprehensive picture of the effect of ASi content on the water holding capacity of soils is still missing.

## 4. Silicon Uptake by Plants

### 4.1. Active Uptake by Intrinsic Transporters

Plant taxa vary in the amounts of Si they take up and accumulate, a variability that is manifested through variations in Si contents, uptake mechanisms, forms and deposition locations. Traditionally, plant taxa have been divided into three major types based on their Si uptake capabilities, defined by the amounts of Si taken up by the plant (often measured as Si content in the xylem) relative to the amounts of available Si in the soil solution. If the amount of Si in the plant is considerably larger than that in the soil solution, the plant takes up Si actively; if the amount of Si in the plant is considerably lower than in the soil solution, the plant excludes Si; if the two contents are comparable, the plant takes up Si passively [104]. As straightforward as this division is, it is over-simplified and lacks mechanistic rigor.

For once, the reference to available Si content in the soil solution can be misleading since the great variability of soil Si pools implies that a species may take up Si both actively and passively, depending on the soil type and parent material. A dynamic approach is more appropriate since it indicates the plant’s response to varying soil Si availabilities and can furthermore point to the underlying internal (physiological) drivers of such responses [7,16]. For example, some species seem to increase their active Si uptake when Si availability in the soil is lower, suggesting an active response to fulfill plant internal Si demands when passive uptake is insufficient [105,106]. In some cases, this is achieved by increasing the expression of Si transporter genes and the density of these transporters under low Si availability conditions [16], indicating a truly active uptake that does not only rely on active uptake mechanisms but also on physiological responses of these mechanisms. Furthermore, Si uptake also depends on transpiration rates, with some species demonstrating passive (transpiration-driven) Si uptake in addition to active (transporter-governed) Si uptake [105,106,107]. The modes and drivers of Si uptake and accumulation and its variability among species are, therefore, not as simple as an active/passive/exclusion division implies.

**Figure 1:**
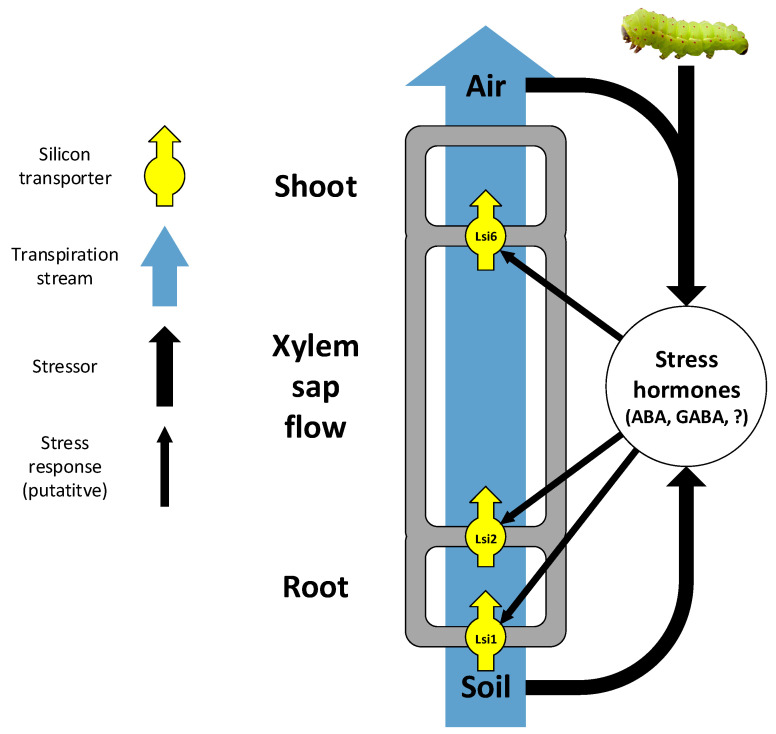
A simplified model of Si uptake from the soil to the shoot through the transpiration stream, including main transporters and responses to external factors.

Several transporters and genes that are involved in Si uptake and accumulation have been studied so far. Although the study of Si transporters focuses on rice and other grasses (as is commonly the case in plant Si research [5,18]), the first plant gene to regulate Si accumulation was discovered in the gourd *Cucurbita* (Cucurbitaceae), regulating Si and phytolith formation in the fruit rind [108]. Shortly after, a surge of discoveries of the physiology and genetics of Si uptake in grasses has arisen, revolving around the four Lsi transporters, all belonging to the NIP aquaporin family. The first transporter to be discovered was the influx transporter Lsi1, located in the distal plasma membrane of root exodermis and endodermis cells [109]. An efflux transporter on the proximal plasma membrane of the same cells, Lsi2, transports Si from the exodermis to the cortex and further loads it from the endodermis onto the xylem [110]. A third transporter, Lsi6, exists in the shoots and is responsible for xylem offloading [111]. In grass shoot nodes, Lsi6 and Lsi3 (previously thought to be Lsi2 due to structural similarities) are involved in distributing Si among branches [112,113,114,115]. Together, these transporters constitute an elaborate cooperative system of Si uptake and distribution in grasses, with some variations in the details of where exactly each transporter is localized within each species [16,112,116] (Figure 1).

Lsi1 and Lsi6 transporters were also identified in the soybean *Glycine max* [117]. In the Cucurbitaceae, a Si-accumulating dicotyledonous family, Lsi1 was also identified in all root cells of *Cucurbita* [118]. Wang et al. [119] identified two putative Si transporters in cucumber (*Cucumis*) of the same family. Together with the early-identified gene responsible for Si accumulation in *Cucurbita* rinds [108], these studies suggest that Si transport systems in grasses and dicotyledons share some similarities. The recent identification of a gene regulating Si uptake by the mangrove *Rhizophora apiculate* without identifying the transporter itself [120]. Multiple genes regulating Si uptake and accumulation were also found in the horsetail *Equisetum arvense* [121]. Finally, it appears that Lsi-like genes that govern Si uptake are common in many groups of land plants, suggesting that the origins of these mechanisms are as ancient as the origins of land plants [122]. These findings further suggest that the physiology and genetics of Si transporters in non-grass species are only beginning to reveal themselves.

Expression of the *Lsi1* gene in rice is downregulated by Si supply, dehydration stress and abscisic acid (more strongly in Si-depleted plants), suggesting regulation of active Si uptake in response to changes in the transpiration stream and plant internal water balance [123]. Further studies have demonstrated how the expression of *Lsi1*, *Lsi2* and *Lsi6* genes is regulated by plant hormones [124] and internal Si and metal concentrations [125,126].

### 4.2. External Factors Affecting Silicon Uptake

In addition to intrinsic transporters, external factors also affect Si uptake and accumulation in plants. These include both passive uptake mechanisms driven by the transpiration stream (the soil–plant–air continuum; Figure 1) and active mechanisms induced or enhanced by biotic stressors. Since Si is taken up from the soil as monosilicic acid within the soil solution, passive Si uptake depends on the transpiration stream. Several studies have shown that plant Si content in grasses increases with soil water content and availability, most probably for the simple reason that the more water a plant absorbs, the more Si is taken up with it [127,128,129,130,131,132,133]. On the other hand, transpiration, acting as the motive force of water uptake, has also been shown to increase Si content in grasses, to the degree that Si content has been suggested to serve as an indicator to plant transpiration stress [113,133,134,135,136,137]. Hence, along large rainfall gradients, Si content tends to demonstrate a U-shaped curve (minimum Si content at approximately 200–300 mm mean annual rainfall), implying an interplay between water availability and transpiration motive force [134,136]. Nevertheless, high plant Si contents in extremely arid conditions may also occur because grasses under drought stress take up Si more actively for the benefit it confers in resisting drought or in herbivore deterrence (see Section 5 below) [134,136,137]. Nevertheless, the failure to observe this positive correlation in other studies of grasses [138,139] suggests that other variables may confound the simple positive effect of water availability [134,136] (For Si effects on soil water availability co-appearing with Si availability in soils, see Section 3.2 above). In the Asteraceae family, an intermediate Si accumulator with mostly passive Si uptake (as far as we know, no attempts were made so far to identify Si transporters in this family), there appears to be no clear, consistent pattern, suggesting that Si uptake is not simply driven by the transpiration stream [134,136]. That the expression of the *Lsi1* gene in rice is down-regulated by dehydration stress and abscisic acid [123] is a further indication for the complex effects of the transpiration stream on Si uptake.

Several studies have shown that ambient CO_2_ concentrations also affect plant Si uptake, content and form, but with contradictory results. Ambient CO_2_ concentrations had no effect on root and shoot Si contents in sugarcane plants [140]. In rice, increased ambient CO_2_ concentrations reduced husk Si deposition by as much as 60% [141]. Increased ambient CO_2_ concentrations alter the composition of phytolith assemblages in *Phragmites* and reduce mean phytolith size [142], suggesting an effect on Si allocation and distribution. Despite these studies being limited and equivocal regarding the regulatory role of CO_2_ on plant Si uptake and accumulation, they harness potential significance for our understanding of global Si–carbon relationships, namely the possibility of Si being a partial substitute for carbon in plants (see Section 5 below) and Si’s role in regulating the carbon cycle (see Section 6 below).

Among the biotic stressors known to affect plant Si content, herbivory is the one that was studied the most [143]. Exposure to invertebrate [144,145,146,147] and vertebrate [144,148] herbivores induces Si uptake and accumulation in grasses. Comparable induction by artificial clipping [149,150,151] further supports that this induction is directly associated with biomass removal or damage. While such induction was sometimes not observed in controlled experiments [144,151,152,153,154,155], this is likely because these experiments did not incorporate sufficiently long exposure times to initiate a response [134,144]. In natural landscapes, higher grass Si contents are associated with larger densities of herbivorous rodents [154,156,157,158,159], but not of larger herbivores [137,148,160] or following clipping [151], which is most likely explained by the involvement of other environmental variables in natural ecosystems having stronger effects on Si uptake and accumulation [134]. Nevertheless, recent evidence for cyclic dynamics of vole densities and grass Si contents [157,159,161] provides further support to the induction of grass Si uptake by herbivory. Among the Asteraceae, the only non-grass family in which the possible effect of herbivory on Si has been widely studied, such an effect was rare and weak [137].

## 5. The Variability of Silicon in Plants

### 5.1. Methods for Extracting Si from Plant Material

There are several methods to quantify plant Si concentration. We describe the six most used methods hereinafter (Table 1), all starting from ground plant material. The easiest extraction method is the method using a 1% Na_2_CO_3_ solution [80,162,163,164], first developed by DeMaster [165]. For this method, ~30 mg plant material is extracted in a 1% Na_2_CO_3_ solution for 5 h at 85 °C, afterward filtered (0.45 µm) and analyzed.

The second method is comparable easy and uses a 0.5 M NaOH solution [162]. For this ~100 mg plant material is extracted in 0.5 M NaOH solution for 5 h at 85 °C, afterward filtered (0.45 µm) and analyzed.

A more complex method is using hydrofluoric acid (HF), which can be seen not only as extraction but as real digestion. Puppe et al. [166] performed a 2-step extraction method using a closed vessel microwave system. For this, they used ~100 mg plant material together with a mixture of 4 mL distilled water, 5 mL nitric acid (65%) and 1 mL hydrofluoric acid (40%) at 190 °C for the first step. In the second step, the hydrofluoric acid is neutralized by 10 mL a 4% boric acid solution at 150 °C and can be analyzed afterward.

Another method used for Si extraction from plant material is the lithium metaborate fusion method [167,168]. For this, the plant material in the porcelain crucible is ashed in a muffle furnace by gradually increasing temperature to 500 °C and holding 500 °C for at least 1 h. Afterward, 5–100 mg of the ash are mixed with lithium meta-tetraborate (1.6 g LiBO_2_ and 0.4 g Li_2_B_4_O_7_) is used to extract the Si at 1000 °C for 5 min in the muffle furnace. The obtained bead is transferred into a 10% nitric acid in a conical flask and stirred at 90 °C until the bead is dissolved. Afterward, the solution is diluted to 100 mL with 10% nitric acid.

Another method to extract plant Si is the tiron method [169], which is comparable with the Na_2_CO_3_ and the NaOH method as the sample is also extracted at 85 °C. For this method, ~50 mg plant material was added to 30 mL of a 0.1 M tiron solution buffered at pH 10.5 and put for 2 h at 85 °C. For this 30 mL, only 10 mL are taken and mixed with 10 mL of 30% H_2_O_2_ and put again for 1 h at 85 °C in order to destroy the tiron, afterward filtered (0.2 µm) and analyzed.

Another simple method is the Si analysis by X-ray fluorescence spectrometry [170], for which no extraction is required, but the Si content in ~100 mg plant material is directly measured, but calibrations are required. Nakamura et al. [168] compared the Na_2_CO_3_ method with the borate fusion method and found that the Na_2_CO_3_ method resulted in lower Si concentration than the borate fusion method. However, the difference can be Na_2_CO_3_ data can be corrected by a simple equation.

### 5.2. Types of Variability

As discussed above, the division of plant species into active Si accumulators, passive accumulators and excluders is not straightforward, and the same follows for dividing taxa into high- and low-Si accumulators. First, a clear Si content threshold does not exist, although an often-cited and widely accepted threshold is 1% Si by dry weight or 1000 phytoliths per g dry weight [23]. Second, although the variability of Si content has a clear taxonomic signal, with orders accounting for 67% of the variation [172], phylogenetic analyses indicate great variations below the order level [23]. For example, most literature cites the grass family Poaceae and more generally the order Poales as being the most Si-rich, at least among angiosperms [18,172,173,174], which is in part why a large portion of plant Si research focuses on this family [5,18]. However, Poales is also the only commelinid order to date in which Si-poor families were observed (e.g., Typhaceae) [23]. In contrast, non-commelinid monocots are generally Si-poor [23,173,174], with some exceptions like the orchid subfamily Epidendroideae [175]. The occurrence of high Si contents in a single subfamily of orchids (one of the largest plant families globally) is not unique: 29 of the 412 angiosperm families include both Si-rich and Si-poor species (7% of all angiosperm families, but 17% of families for which data exist) [23]. Finally, it should be noted that although most research revolves around seed plants, silicon and phytoliths are common and abundant in many bryophytes and pteridophytes [122,172,176,177,178] (phytolith-like “mycoliths” also occur in fungi [179]), sometimes in contents that surpass those found in Poaceae species [172].

**Figure 2:**
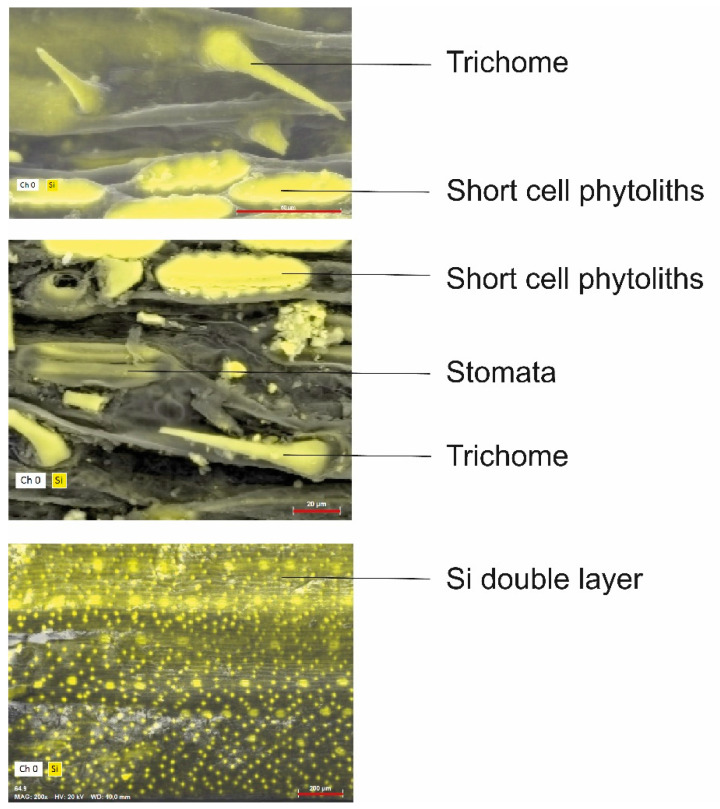
Different Si deposits occurring in the leaves of grasses. Here SEM-EDX pictures of wheat left at maturity. The red line indicates the scale for the different pictures (50 µm top, 20 µm middle and 200 µm bottom picture). The SEM-EDX analysis results were provided by J. Busse (ZALF, Germany).

After its uptake by the roots, monosilicic acid is transported up to the shoot via the xylem, with the transpiration stream acting as the main motive force [11,16,106,135]. Within the plant tissues, large quantities of monosilicic acid begin to polymerize, first into polysilicic acid and further into ASi [180,181,182,183]. Some organic molecules, such as amines, amino acids and prolines, have been shown to be involved in this process [180,181,182,184,185,186]. There are many forms of ASi in plants [183,187] (Figure 2), most notably the cuticle–Si double-layer [41,53,188], cell wall-bound Si [185,189,190,191,192,193,194], and phytoliths that develop inside cells [63,68,69,70,173,174,195]. It is unclear whether some of these forms are deposited earlier or at higher rates than others, but variations in their condensation states suggest different formation processes or rates and probably different functions [196]. Some scholars use the term “phytoliths” in a narrow sense to describe ASi formed in short epidermal cells (sometimes called “silica cells” or “raised platforms”) and trichomes, while others use it in a broader sense to describe any type of plant ASi formed in direct association to the cell, including long epidermal cells (whose phytoliths are often articulated into “silica skeletons” [133]), trachea, etc. [174,197]. Here, we shall use the broader meaning of the term and specify any particular phytolith subtype when appropriate.

The distribution of Si among these forms is variable and disputed. While early accounts suggest ASi (mainly phytoliths) constitutes 90% of plant Si [58,198], more recent analyses have suggested that ASi constitutes 15–79% of plant Si, the remainder being mostly polysilicic acid [199]. Despite several decades of studying Si deposition mechanisms, we still have a very partial picture of the topic, focusing on grass species and mostly on their prominent and diagnostic epidermal silica short cells. Blackman proposed one of the earliest developmental frameworks for Si deposition in grasses, which consisted of two main observations, which have been further corroborated by later studies. First, Si deposition does not take place in all cell and tissue types at the same timings and rates. “Typical” silicification of specialized cells (e.g., short epidermal cells) is constitutive and occurs under all growth conditions and precedes facultative “atypical” silicification of adjacent unspecialized cells (e.g., epidermal long cells and stomatal cells), which possibly depends on sufficient amounts of water, Si and transpiration [63,68,200,201]. Second, some cells (mainly epidermal short cells) undergo differentiation to become specialized Si-accumulating cells and then undergo further anatomical and physiological changes (e.g., lignification and apoptosis) to accommodate Si deposition and filling [69,191,195,202,203]. Later studies have shown that Si deposition first takes place in the cell wall or other external parts of the cell, and Si filling of the cell lumen takes place only later [191,202,204].

Silicon contents also vary among plant parts. Most evidence suggests that epidermal tissues are the most Si- and phytolith-rich and thus that plant parts with large surface areas (namely leaves and inflorescences) are more Si- and phytolith-rich than other parts like stems and roots [59,60,174,205,206,207,208,209,210,211]. However, this is not always the case. For once, while most evidence comes from grasses, other herbaceous and woody species present different patterns. In some woody species, phytolith (which are commonly the most abundant Si form in plants [58,198,199]) occur in larger amounts in wood and bark than in leaves [174] (but see [209]), and in the forbs stem, Si contents also exceed those in leaves [205,212]. Inflorescences commonly have more Si than leaves or other vegetative parts in grasses [59,60,137,210,213], but this is not always the case in some grasses [208,209,210] or in forbs [137]. Many studies have also shown that Si content in roots is lower than in aboveground plant parts [60,174], but again with some mixed observations [214]. Understanding the sources of differences among plant parts and how these patterns vary among plant taxa may prove to be a complex task. Epidermal tissues are more Si-rich because they are terminal points of the transpiration stream and hence preferable sites for Si deposition, but many plants actively direct Si deposition into these tissues and plant parts [69,191,195,202], and this deposited Si plays certain roles that are related to its position (see Section 5.2 below).

These three types of variability (among taxa, among forms and among plant parts) are further manifested—albeit in somewhat different manners—in the variability of phytolith morphologies and morphological groups (morphotypes). This variability is by far the one that is best documented and analyzed [197,215,216,217,218,219], in part because of its usefulness in palaeoecology and archaeology [131,132,133,174,208,209,210,216,220,221,222,223,224,225,226,227,228,229,230]. Of no less importance, but less developed is the potential of using morphotypical analyses in studying plant evolutionary history, owing to the correspondence of some morphotypes to phylogeny and thus the ability to identify plant taxa ancestors using putative ancestral morphotypes [176,195,231,232,233].

### 5.3. Form, Location and Function

Despite all the amounts of data and knowledge of the variability of Si in plants, it appears that this knowledge tends to be patchy. This should come as no surprise since the many studies over the years had different questions and purposes. However, this leaves us with a big question: is there a pattern connecting form, location and function? We are capable of partially answering this question since we do know, for example, that Si-rich grasses accumulate Si mostly as phytoliths in aboveground epidermal tissues, which probably plays certain functions. Nevertheless, while evidence for each type of variability is relatively ample, there is only meager evidence for their coalescence into clear patterns (syndromes) that have clear physiological, developmental or ecological implications. If such syndromes exist, then different Si forms in different plant parts probably have different functions in different taxa and thus reflect physiological and developmental pathways that further have ecological and evolutionary implications [1,18,234,235]. For example, do taxa with different Si forms or that accumulate Si in different parts, or tissues do this because they have different Si uptake and allocation mechanisms? Do some forms occur earlier, more often or in larger quantities in some plant parts or tissues than others do and is this driven by any internal or external factor? If different syndromes are found in different taxa (or, not less interestingly, if not), what can we learn from it about the evolution of this phenomenon or the mechanisms that govern it? Furthermore, connecting to the Si functions in plants and its soil chemistry, what can we learn from this about the role of Si in shaping ecosystems and about ecosystem management, or even about shaping and managing the ecosphere as a whole?

**Figure 3:**
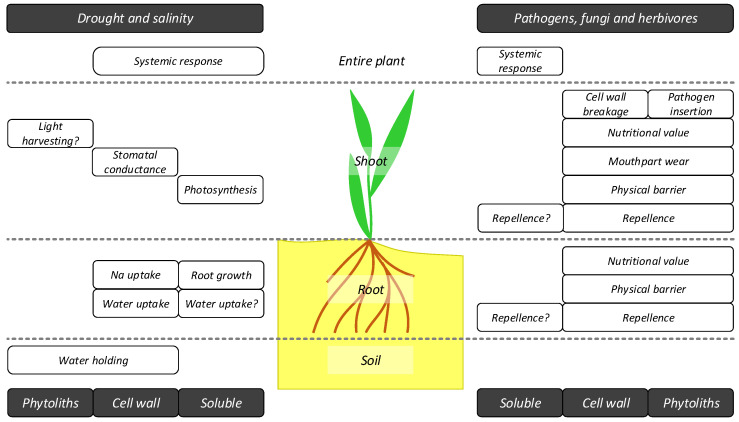
Form, location and function of Si in plant protection from drought and salinity (**left**) and from pathogens, fungi and herbivores (**right**).

Silicon protects plants from drought stress through many mechanisms [128,236,237] (Figure 3), known mainly from grasses. Many of these mechanisms are also relevant for salinity stress alleviation, owing to the multiple similarities between the two stresses [236,237,238,239], but we shall focus on drought hereafter. Silicon promotes root growth and improves water uptake by root, allowing plants to maintain stomatal conductance [128,236,240]. While the mechanism and the form of Si involved in this are not fully understood, it is probable that Si deposition in the endodermal cell wall is at least partially involved in improving water uptake [241]. Silicon in the cell wall is also involved in regulating stomatal movement and conductance, hence regulating water loss through stomata [193,242,243,244]. The sub-cuticular Si layer, which has long been suggested to play a role in water loss reduction [198,245], was recently found to reduce water loss from the cuticle by as much as 23% [246] (but see [242], who found no effect on water loss from the cuticle). Although most leaf surface water loss takes place through stomata [247], this 23% reduction can be significant. Silicon—most probably in a soluble form—activates several internal physiological mechanisms that signal and alleviate oxidative damages caused by drought, including increasing photosynthesis rates [248,249,250,251,252,253]. The idea that epidermal phytoliths act as prisms that harvest more light into the mesophyll has little support [254]. Finally, ASi in the soil has been shown to improve water holding capacity and thus water availability to plants [73,88,101,103], possibly due to its hygroscopic properties [102]. Albeit not an in planta process, plants play a key role in the Si cycle and large quantities of soil ASi come from plant litter or herbivore feces [21,22,23,171,255,256,257,258,259,260,261,262,263,264,265], so this is plants’ contribution to their offspring and neighbors, and hence an indirect function through soil appropriation, facilitation and possibly even ecosystem engineering (see Section 6).

The function of Si in protecting plants from pathogens, fungi and herbivores (Figure 3) includes protection in various stages, from repellence to penetration prevention, to damage to herbivore performance during and post-ingestion, and finally to activation of physiological responses to damage. The type and magnitude of this protection vary among herbivores, depending on their size, feeding guild and Si forms to which they are exposed [235,266]. A repellence function is manifested mainly in studies of invertebrate and vertebrate herbivores, which often prefer Si-poor over Si-rich plant food [140,144,149,267,268,269] (for a parallel role in sponges, see [270]). It is likely to include both physical repellence by silicified trichomes and short epidermal cells (Figure 2) that increase leaf surface roughness [271], but other mechanisms may also be involved. If repellence does not occur or is not sufficiently effective, Si can prevent pathogens and fungi from penetrating the plant body. This function is fulfilled by ASi (both as epidermal cell phytoliths and within the cell wall) [271,272,273,274,275,276,277]. However, there is no empirical evidence for a similar function of the Si-cuticle double layer, contrary to some suggestions [278,279]. Comparably, abrasive ASi can damage invertebrate [267,280,281] and vertebrate [282,283] herbivores’ mouthparts (but see a critique of tooth wear by phytoliths in [284,285]). Furthermore, it is possible sharp phytoliths (e.g., trichomes, Figure 2) may also insert pathogens into herbivores’ soft tissues [286]. However, it is unknown at what stage of their development these ASi deposits become sufficiently condensed/rigid to affect herbivores and whether different degrees of condensation/rigidity function differently [196]. While ASi abrasiveness can have a negative effect on herbivores, this effect is probably long-term [280] and does not have an immediate negative effect on herbivores. Nevertheless, once ingested, large quantities of ASi can impair herbivore nutrition through two separate mechanisms. Quantitatively, large ASi contents in food come at the expense of other nutritional components and can even modify plant chemical composition [85,207,287,288,289]. Qualitatively, cell wall ASi hinders cell wall breakdown in animal intestines, reducing the released amount of chlorophyll and other nutritious compounds that can be digested by herbivores [290]. These two mechanisms, with the addition of low preference to Si-rich plant foods, often results in reduced herbivore growth [140,146,267,272,280,282,288] and even cause a herbivore population decline [157,159,161]. There is even some evidence that plant Si can cause urolithiasis [58,291,292,293] and be carcinogenic [294]. Finally, Si—most probably mainly in its soluble forms, induces several systemic defense mechanisms [147,295,296,297,298,299,300] (but see [301]), including attracting the herbivore’s natural enemies [145]. Notably, apart from a handful of studies dedicated to roots [140,147], all other studies focused on plant aboveground parts (mostly leaves) or on systemic responses that also tend to use information from aboveground parts.

The multiple physical, chemical and physiological Si plays in plant life in its various forms (Figure 1), and the benefits they appear to confer on plants, have arisen an intriguing question in recent years: is Si a partial substitute for carbon [1,2]. The credit for first suggesting this idea goes to Raven, who in 1983 estimated the metabolic costs of Si-based mechanical support are10–20 times less than the metabolic costs of lignin [11]. Several later studies have shown that plant Si content trades off with plant lignin and cellulose [302,303], phenols [7,207,304,305,306,307], and some nutrients’ [207,287,289,307] contents. Moreover, grasses accumulate less Si at higher atmospheric CO_2_ levels [141,306,308]. However, no such effects of atmospheric CO_2_ were found in sugarcane [140] and Si-rich trees [309]. There is also no consistent difference in Si content between C_3_ and C_4_ grasses [310], which differ in their carbon use efficiency. Therefore, and also considering the lack of global-scale analysis, the case for a universal tradeoff remains disputed at this point [1].

## 6. Implications for Ecosystem Structure, Functioning and Services

Although the many functions Si plays in plant biology and ecology have been recognized for many years, it is only within the past decade and a half that this understanding culminated in recognition of plant Si uptake as an important phenomenon in ecosystems and as a plant functional trait [1,2,25,311].

### 6.1. Effects on Soil

#### 6.1.1. Si Cycling in Undisturbed and Disturbed Plant–Soil Systems

**Figure 4:**
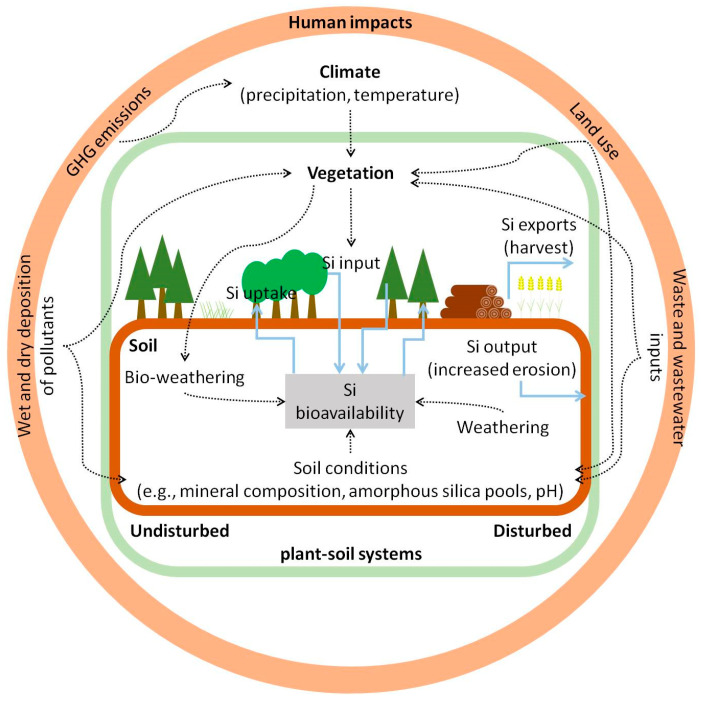
Schematic overview of Si cycling in undisturbed (unused, natural) and disturbed (used) plant–soil systems. Si bioavailability in soils, and thus Si cycling, is strongly influenced by human impacts, i.e., greenhouse gas (GHG) emissions, land use (agriculture, forestry), pollution via wet and dry deposition, and waste and wastewater inputs.

Mineral weathering represents the ultimate source of Si in terrestrial ecosystems and thus controls Si concentrations in soils. Weathering in soil–plant systems, in turn, is controlled by climate (precipitation, temperature), specific soil conditions (e.g., mineral composition, quantity and physicochemical properties of amorphous biogenic silica, soil pH), and vegetation (Si uptake and recycling) [312,313,314] (Figure 4). In general, dissolution of minerals is much slower than the dissolution of amorphous (biogenic) silica like phytoliths, i.e., phytoliths are 10² to 10^4^ times more reactive than clay minerals and primary silicates under common soil pH (about 4 to 8) [315,316]. Consequently, the (re)-cycling of Si by organisms, especially plants, has gained much attention as it strongly influences the Si cycle on a global scale, especially by accelerating Si turnover rates and export to riverine and marine systems [259,317].

Bioavailable Si is accumulated to a large extent in several major biome types, e.g., forests (11.7 Tmol y^−1^), steppes (13.3 Tmol y^−1^), and cultivated lands (29.4 Tmol y^−1^) out of a total of 84 Tmol y^−1^ for all terrestrial biomes [21,256]. However, humans directly affect the distribution and size of these biomes and thus influence corresponding Si cycling through intensified land use, i.e., forestry and agriculture (changes of soil properties and vegetation) [318,319,320]. Additionally, increased greenhouse gas emissions and consequential changes in climate conditions may have severe impacts on Si cycling [258]. Clymans et al. [321] estimated that the total amorphous (biogenic plus minerogenic) Si pool in temperate soils decreased by about 10% within the last 5000 years due to human land use. Amorphous silica, in turn, has been found to increase the water-holding capacity of soils (see Section 3.2 [103]), influence nutrient supply (e.g., phosphorus and organic matter mobility [81,83]) and act as the main source for bioavailable Si [166,322].

Concentrations of amorphous Si are considerably lower in agricultural soils than non-agricultural soils, e.g., forest or steppe soils. This is because Si exports through harvested crops generally lead to loss of Si in agricultural plant–soil systems (i.e., anthropogenic desilication) [20,323,324,325,326]. However, some agricultural practices might also increase Si availability in soils, e.g., human set fires [327], the application of Si-rich fertilizers [26], or liming (pH effect, [328]). On a global scale, about 35% of Si accumulated in vegetation is synthesized by field crops, and this proportion is going to increase with increased agricultural production within the next decades [256]. Si uptakes, which can be assumed to equal Si outputs by harvesting, of cereal crops are quite high and reach up to several 100 kg per hectare in a year (e.g., rice: 270–500 kg Si ha^−1^ y^−1^ [20]; 230–470 kg Si ha^−1^ y^−1^ [329]; sugarcane: 379 kg Si ha^−1^ y^−1^ [330]; wheat in the temperate zone: 20–113 kg Si ha^−1^ y^−1^ [20]).

In contrast to natural ecosystems (Si uptake of 2–127 kg Si ha^−1^ y^−1^ [20]), where large amounts of Si are recycled year by year [322], the annual Si exports in agricultural soil–plant systems are mostly not compensated. However, targeted manipulation of Si cycling (e.g., Si fertilization, straw recycling) might be a promising strategy to both prevent desilication of agricultural plant–soil systems and consequently improve crop resistance against abiotic and biotic stress (see Section 5.3), and enhance carbon sequestration in agricultural biogeosystems to mitigate climate change [331,332,333]. Carbon sequestration in agricultural systems may be enhanced by the regulation of weathering (e.g., silicate rock powder amendment), organic C stabilization (e.g., silicon and biochar fertilization), and phytolith-occluded carbon (e.g., partial straw retention after harvest) [333] (Figure 4). However, it should be noted that the potential of phytoliths in C sequestration is still under controversial discussion (see the review by Hodson [334] and references therein). However, this carbon occlusion is potentially the carbon remaining from the protein template shaping the phytoliths [184].

Guntzer et al. [324] analyzed archived soil and plant samples of the long-term Broadbalk Winter Wheat Experiment at Rothamsted Research in the UK. They found that the long-term removal of wheat straw considerably decreased amorphous silica pools in soils. However, they did not observe a distinct relationship between the decrease of amorphous silica and a corresponding decrease in Si concentrations of crop straw. In fact, Guntzer and colleagues [324] found such a relationship only for the samples taken before the year 1944. After this year, Si concentrations in straw tended to increase. From their results, Guntzer et al. [324] concluded an increased soil pH due to periodic liming to increase amorphous silica (i.e., phytoliths) dissolution, and thus to represent the main driver of increased Si uptake by the cultivated wheat plants. This is underpinned by a recent study of Caubet et al. [335], who ascribed an increase of calcium chloride extractable Si in cultivated soils (perennial and annual crops) in France to liming. However, it must be kept in mind that Si availability in (agricultural) soils is determined by a complex interaction of factors, and thus liming effects on Si availability follow no general rule, i.e., there are studies showing negative and other studies reporting positive correlations between pH and Si availability (see the review by Haynes [328]).

A long-term field experiment (established in 1963) in NE Germany revealed that about 43–60% of Si exports could be saved by crop straw recycling [336]. These authors found crop straw recycling to become more effective the longer straw recycling is applied, indicated by an increase (or replenishment) of plant-available Si in soils with time, which was also reflected by increasing phytolith contents in these soils. In fact, plant-available Si increased from about 5 mg kg^−1^ (a value that is comparable to other agricultural sites in the temperate zone [337]) to about 10 mg kg^−1^ (comparable to undisturbed ecosystems like forests under temperate conditions, e.g., 10–40 mg kg^−1^ [338]; 7–40 mg kg^−1^, [339]; 4–80 mg kg^−1^ [337]) within 42 years of straw recycling [336]. Thus, straw recycling in combination with soil [340] and foliar [341] Si fertilization might be the most promising strategy to restore natural Si recycling processes in agricultural ecosystems to the highest possible extent and produce resilient crops in modern, sustainable agriculture.

Klotzbücher et al. [342] analyzed Si mobility in soils depending on sorption competition with highly competitive compounds (i.e., dissolved organic matter and phosphates). From their results, they concluded a weaker binding strength of Si (as compared to P and dissolved organic matter) to Fe oxides leading to Si mobilization by the input of P and dissolved organic matter. However, it should be kept in mind that the laboratory experiments of Klotzbücher et al. [342] were performed under acidic conditions (pH 4), resulting in a potentially decreased binding strength of Si due to slowed polymerization of H_4_SiO_4_ [26]. According to these findings, it can be assumed that mineral fertilization, as well as organic matter input by crop straw recycling, should increase Si mobilization in soils resulting in potentially higher Si accumulation in plant biomass [20]. Li et al. [343], for example, found combined Si–P fertilizers to increase concentrations of plant-available Si in soils leading to higher biomasses and phytolith contents of rice plants. However, because Si–P interactions in the soil–plant system are driven by complex biogeochemical processes that are still not fully understood [83], further studies are needed to shed light on this aspect. In this context, it is of great interest to study to what degree Si uptake of cultured plants is determined by their phylogenetic position and environmental factors like temperature or Si availability [172,173,304].

Miles et al. [344] analyzed 28 sites located throughout the sugarcane-growing areas of South Africa and found a close correlation between plant-available Si in soils and Si contents in sugarcane leaves. Regarding rice production, Korndörfer et al. [345] analyzed 28 field experiments in the Everglades Agriculture Area, representing a wide range of available Si in soils. They found plant-available Si in these soils to be correlated with Si contents in rice straw. In contrast to these studies, which considered several study sites with relatively large gradients in plant-available Si, Klotzbücher et al. [303] found no relationship between plant-available Si in soils (herein concentration of dissolved Si in soil solution) and Si contents in rice straw in one (i.e., the drier one) of two analyzed cropping seasons in a field experiment in Southern Vietnam. However, they found such a correlation in the second cropping season, i.e., the wetter one. From their results, Klotzbücher et al. [303] speculated climatic differences to be responsible for their observation (cf. [346]) and concluded field experiments to be inconsistent with results from laboratory studies regarding relationships between plant-available Si in soils and Si uptake by plants (e.g., [347,348]). This is underpinned by a study by Keeping [349], who found that the uptake of Si by sugarcane in a shade house pot experiment did neither reflect the concentration of plant-available Si in soils nor the Si content of used Si sources (calcium silicate slag, fused magnesium (thermo) phosphate, volcanic rock dust, magnesium silicate, and granular potassium silicate).

Non-agricultural soils like forest soils are characterized by a soil horizon-related distribution of amorphous silica showing the highest concentrations in the organic horizons (dominated by phytogenic silica) and a decrease in the deeper mineral horizons [322,337,350]. However, bioturbation and percolation can affect the distribution of amorphous silica [171,339,351,352]. The concentration of phytogenic silica in forest soils mainly depends on the quantity of plant materials, i.e., litterfall and other plant residues supplied to soils (phytolith input) and the loss of phytoliths (phytolith output) via harvesting of trees, erosion (wind, water), translocation (bioturbation, percolation), and dissolution (e.g., [22,258,312,313,353]). In this context, it should be kept in mind that phytolith inputs are not only driven by aboveground plant materials but also by plant roots [354,355]. Physicochemical properties of phytogenic silica (phytoliths) control their susceptibility to dissolution, and these properties differ between fresh and aged phytoliths with implications for Si availability in soils [356]. Changes of the dominating vegetation by humans (deforestation) are leading to large Si exports declining the concentration of amorphous Si in soils [318]. Moreover, increased erosion [318] or human-set fires [327] have the potential to alter amorphous silica concentrations in soils, and thus Si availability. The depletion of pedogenic Si pools in the long-term, i.e., during ecosystem retrogression, might even increase the importance of Si recycling by plants [357]. Finally, plants themselves can actively increase Si bioavailability in soils by increasing soil weathering in the rhizosphere (bio-weathering, see [358,359,360].

#### 6.1.2. Concluding Remarks

Regarding biogenic amorphous Si in soils, it should be kept in mind that the vast majority of studies have been focused on phytogenic silica, i.e., phytoliths (cf. [26]). However, the importance of other biogenic Si pools, especially the protozoic one (represented by a group of protists, i.e., testate amoebae), for Si cycling in some ecosystems has been revealed, and their significance for Si cycling in terrestrial ecosystems might be comparable to the role of protists (i.e., marine diatoms) for Si cycling in the oceans (see the review by Puppe [361]). Recent studies indicate that protozoic Si pools are strongly affected by land use [362,363], but we still do not know which effects protozoic Si pool changes have on the ecosystem scale (e.g., impacts on Si availability). Furthermore, we still do not know if these organisms are able to increase weathering rates in soils by bio-weathering. In fact, there are some hints that bio-weathering may play a role in protists [364], underpinning their potential significance for Si cycling in terrestrial ecosystems.

The bioavailability of Si in soils is controlled by at least three key factors: (i) the Si concentration in soil solution, (ii) the reserve in the solid phase as Si source (minerogenic/pedogenic, biogenic, adsorbed, or fertilizer Si), and (iii) the Si adsorption capacity or retention capability of the soil [328,365]. As all of these factors are the result of complex biogeochemical interactions and thus differ from one soil to another, a general understanding of Si availability in different soils and its uptake by plants and other organisms represents a hard-set challenge. Unfortunately, there is no standard extraction method for the determination of Si availability in soils yet (see [26] for more details), and thus different studies often show inconsistent results. Crusciol et al. [366] showed that correlations between plant-available Si in soils and Si concentrations in sugarcane were not only dependent on soil texture but also on the used extractant (i.e., CaCl_2_, deionized water, KCl, sodium acetate buffer at pH 4.0, and acetic acid). In fact, there is no common standard procedure for the evaluation of plant-available Si in soils because these procedures have been developed for specific plants in specific climates, i.e., mainly sugarcane and rice in (sub)tropical zones (cf. [72]).

What we need now is detailed research on methods for a reliable determination of Si availability in soils of undisturbed (natural) and disturbed (used) plant–soil systems (Figure 4). In this context, a combination of (i) different information on the Si status of soils (e.g., the concentration of extractable plant-available Si, quantity and quality of solid biogenic and pedogenic Si phases, retention of plant-available Si in soils, the influence of climate and vegetation) and (ii) laboratory and (long-term) field experiments (e.g., identifying of drivers of plant-available Si in different soils, balances of Si cycling in plant–soil systems) may be the most promising approach to enlighten the complex interactions in biogeochemical Si cycling. Such knowledge is crucial for the understanding of ecosystem structure, functioning, and services.

### 6.2. Effects on Species Interactions, Community Structure and Net Primary Productivity

Due to the multiple functions Si has for plants and the benefits it confers on them, it is only natural to conclude that it has implications on plant fitness, interspecific plant–plant and plant–animal interactions, and hence also on ecosystem structure and functioning [1,2,24,25]. While there is increasing evidence for some of these implications and for the overall large-scale story—as we describe here—the mechanisms and phenomena are often not well understood and sometimes disputed. For a start, clear evidence for the effect of Si on plant fitness (i.e., reproductive success and its contribution to the next-generation) come mainly from grain/seed yield measurements carried out in agricultural studies, which aim to assess Si’s contribution to crop production (e.g., [10,85,253,367,368,369,370,371,372]). Apart from focusing on crops rather than on a broader range of plant species, the application of these measurements for ecological fitness assessment requires information about seed germination and seedling survival. Moreover, for understanding the effects of Si on plant–plant interactions (namely, competition), let alone on plant community composition and its dynamics, a species’ fitness needs to be converted into relative fitness (i.e., relative to neighboring species). Evidence for Si affecting interspecific competition is meager from a single study of competition between two grass species [152]. Understanding how Si affects interspecific competition can be useful for crop growing, especially if the benefits of Si to a crop are smaller than its benefits to competing weeds.

By far, the most intensively studied effects of Si on plant interactions are those that focus on plant–herbivore interactions. We have already discussed the effects of plant Si uptake and accumulation on herbivores (see Section 5 above and Figure 3), but no less intriguing are the effects in the opposite directions. Among the early indications for herbivory affecting plant Si content come from field studies in the 1980s, which have shown that grasses that grow near rodent colonies had higher Si contents than those away from the colonies [150,151,154,156,373,374]. For once, continuous severe herbivory is likely to select in favor of plants that possess anti-herbivory defenses. In those species and cases where Si accumulation plays an anti-herbivory role, it should be no different [137]. Some studies show that under heavy grazing, grasses possibly prefer habitats with higher soil Si availability [150,151,374], which may suggest selection in favor of more Si-rich habitats under herbivory stress [143]. However, this correspondence may be circumstantial or a result of long-term effects of herbivory on soil Si availability (see Section 6.3 below). Moreover, evidence for the evolution of plant Si accumulation as being driven by herbivory is limited and disputed [23,235,375,376].

A better supported, more immediate effect is the induction of plant Si accumulation by herbivory [143] (evidence for a comparable induction by pathogens and fungi was also observed [275,377]). Several studies have documented the induction of Si accumulation following herbivory in grasses [144,150,157,159,374]. Nevertheless, the degree of induction varies among herbivores [134,144,148] and plant genotypes [148,152,160]. A lack of induction in some studies in which defoliation was simulated by clipping suggests that at least part of the variation in effects of different herbivores is connected directly to how the herbivore consumes plant biomass, with saliva possibly being an elicitor [144,150,151,374]. However, it is also possible that induction does not occur in some laboratory experiments [144,151,153,154] because of insufficiently long exposure periods in controlled experiments [134,144]. Moreover, it is possible that prolonged (millennial-scale) heavy grazing selects in favor of constitutive Si accumulation [137]. Among non-grasses, evidence for such an induction—or at the very least to a variation in Si content related to herbivory intensity—is rare and may be coincidental [134,137]. One important outcome of the reciprocal interaction between plant Si accumulation and herbivory is a negative feedback loop of herbivore population size and grass Si content, augmented by delayed responses [157,159,161,378]. According to this model, increases in herbivore population sizes select for higher plant Si content, which in turn reduces food quality and causes a decline in herbivore population size, hence removing the selective pressure from the grasses and allowing their Si contents to decline.

The variations among species in plant Si contents and its responses to external variables (e.g., aridity and herbivory) also suggest that plant Si content affects plant community composition and structure [1,379]. To date, there have been no studies that demonstrate such effects directly. However, Plant community composition is clearly associated with Si and Ca pools and the interaction between them [380]. Moreover, in a long-term plant diversity manipulation experiment, plant Si has been shown to play a role in driving consumer community composition [379]. Schaller and colleagues [381] have proposed a model for plant community composition and biomass production based on soil Ca/Si ratios. Moreover, in a broad-scale study, plant Si content was found to have some effect on ecosystem structure, and most prominently on its herbivore component [379]. On a global scale, biomes with higher plant Si contents tend to have higher net primary productivity (NPP), even when forests and open-habitat biomes are analyzed separately [21]. Concrete evidence for plant Si playing a role in governing community and ecosystem structure, and furthermore ecosystem functioning, do not exist yet, and our understanding of such a role are based almost solely on models and correlations. Nevertheless, the theoretical knowledge points strongly to this direction, stimulating discussions and attention to the issue, so it is probably not long before empirical evidence follows.

### 6.3. Effects on Biogeochemical Cycles

#### 6.3.1. The Ecosystem Scale

The known and expected effects of plant Si on ecosystem structure and hence on their functioning (e.g., NPP), as manifested on a global scale, suggest that this phenomenon also affects biogeochemical cycles. The obvious of these is the Si cycle. Plants recycle large amounts of Si globally, ranging from 2 to 8 t dissolved Si km^−2^ y^−1^ from grasslands, forests and saltmarshes [255,262]. Much of this Si is eventually exported to rivers, lakes and oceans [21,25,255,258,382,383,384,385]. In a study from Hawaii, for example, Derry et al. [384] estimated that as many as 68–90% of dissolved Si in stream water passed through plants. Nevertheless, Si does not affect the Si cycle only but also the carbon, nitrogen and phosphorous cycles. Some of the mechanisms underlying these effects at the smaller scales (e.g., individual plants to ecosystems) are known. We shall focus here on five aspects: effects of Si on plant stoichiometry and litter decomposition, carbon and nitrogen occlusion in plant Si and phytoliths, Si cycling (with emphasis on the roles of weathering and herbivory), and the possible coupling of the Si and carbon cycles.

As Si is increasing nutrient availability in soils (e.g., phosphorus and nitrogen) [81], it is not surprising that the nutrient status of plants is also improved due to Si [85,386]. Si is competing with nutrients for binding sites on soil minerals, and this increases their availability for plant uptake. The same effect is suggested to occur within the plants, with Si potentially increasing nutrient mobility in plants too, and also potentially increasing expression of P transporter genes [387]. These positive effects of Si and plant nutrition are not only shown for grasses [85,207,388] but also for legumes with Si increasing N-fixation by rhizobacteria [389]. However, as N fixation being increased by better P nutrition [5,390] and Si increasing plants’ P nutrition (as shown for grasses, see above), the positive effect of Si on N fixation may be indirectly due to Si increasing plants P nutrition. For sugarcane, no positive effect of Si on P nutrition was found [391]. In contrast, Xu et al. [91] found no positive effect of Si on N in legumes. Overall, the positive effect of Si on plants’ P status seems to be much clearer than the effect of Si on plants’ N status. Another main pattern is the decrease of C concentration with increasing Si concentration in plants, which was suggested as a “carbon partial substitution by Si” in the 1980s by Raven [11]. This pattern of decreasing C with increasing Si concentrations in plants was found in numerous studies [85,207,303,304,388]. This is, of course, no substitution of C atoms by Si atoms in terms of function, but more a specific volume of plant tissue that is filled with Si compounds instead of C compounds.

Due to the changes in plant nutrient status—especially for C, N and P—the relation and stoichiometry between those elements also changed. As C concentration for wheat is decreasing due to Si accumulation and P concentration increasing, the resulting number for the C/P ratios is lower [85]. At constant N concentration, by increasing P concentrations in wheat due to Si, the N/P ratio is also lower [85], although no clear pattern of Si effects on stoichiometry was found in sugarcane [392]. A large study revealed a negative relationship between Si and N/P ratios for wetland and submerged species and a negative relationship between Si and both C/N and C/P ratios for submerged species [289]. A study of the effect of Si fertilization on sugarcane found an increase in the C/N ratio by Si for different cultivars and a decrease in the C/P ratio for one cultivar [391]. A study analyzing grass species in China found an increase in C/N ratio with increasing Si and a decrease in both C/P and N/P ratios with increasing Si [393].

With the changes in plant nutrient concentration by Si, an effect on plant litter and organic matter decomposition is possible. It was shown that leaf material from reed [394,395] and rice [348,396] with high Si concentration decomposed faster than a litter of conspecific plants with a lower Si concentration. However, Emsens et al. [397] found no effect of plant material Si concentration on decay rate. Increased respiration of organic matter with increasing Si availability was found for arctic soils [83] and peat [81]. Most recently, it was shown that the effect of Si stimulating respiration of organic matter is only occurring under oxic conditions, whereas under long-term reduced conditions, no effect was found [398]. This effect of Si increasing soil respiration under oxic conditions may be explained by the fact that Si increases P concentrations in soil pore water [83,398,399] and with this the nutrient availability for the microbial decomposer community. Furthermore, it was shown that an increase of Si in soil pore water might also be able to mobilize carboxylic groups from soil organic matter particles, hence potentially additionally increasing litter decomposition [399]. Another potential explanation for how Si is accelerating organic matter respiration is by a change in soil microbial decomposer community, as shown for reed litter decay—where Si reduced the concentration of ergosterol as a measure of sporulating fungi [395]—and for rice litter—where the abundance of increase in saprotrophic fungi increased with increasing Si availability [396]. Hence, how Si is altering the soil microbial decomposer community is currently unclear, as the existing studies partially contradict each other.

#### 6.3.2. The Global Scale

Since the formation of phytoliths, especially in “typical” specialized epidermal silica short cells, is a controlled process of cell modification, apoptosis and complete lumen filling [69,191,195,202,203,204], it often results in the occlusion of carbon- and nitrogen-rich organic remnants of cell components (presumably from the nucleus) within the phytolith [184,189,333,400,401,402,403,404,405,406,407,408,409,410]. Some studies indicate that at least some of these carbon- and nitrogen-rich organic compounds do not originate in the host cell but are taken up directly from the soil via the roots (and therefore, are “old carbon” that cannot be used for carbon dating) [409,411,412,413,414,415]. Further analyses of these organic inclusions indicate that they contain amino acids but not DNA, which seems inconsistent with the idea that they originate in the cell nucleus [410,416]. This carbon can potentially be preserved within the phytoliths for millennia or more but might be more susceptible to post-depositional oxidation than thought [410]. Some scientists argue—based on theoretical models—that this occlusion has an appreciable impact on carbon sequestration [333,400,401,417,418,419], but it probably does not account for more than 2% of the total terrestrial carbon sink [1,400,420].

An important process in the Si biogeochemical cycle is the weathering of silicate minerals. These minerals vary in their dissolution rates and conditions, from the durable slow-weathering primary silicate minerals to the more labile and fast-weathering ASi [76,315,316,421,422] (there are also differences in ASi dissolution according to plant type [421,423]). Plant Si plays an appreciable role in this process by two main mechanisms. First, plant Si that is reincorporated in the soil through plant litter is a significant soil Si pool, which consists of various Si forms with different physicochemical properties and dissolution dynamics [315,316,322]. For example, the Si double-layer dissolves faster than epidermal silica short cells [395]. Even among phytoliths, some morphotypes are more soluble than others [424,425,426]. Second, Si uptake by plants imbalances the soil equilibrium of different Si forms (it removes soluble Si and reincorporates it into the soil as ASi), and thus promotes re-equilibration through silicate mineral weathering [22,171,256,263,359,424], especially if some of the plant Si is exported by herbivores, farmers, etc., [20,260,422]. It is possible that this process is often accelerated by plant excretion of organic acids that dissolve mineral Si in order to meet plant Si demands (cf. [359]).

Herbivores are now acknowledged as being one of the three main agents of nutrient cycling, alongside decomposition and fire [427,428]. Likewise, herbivores that consume large amounts of Si-rich plant biomass (e.g., grazers) play an appreciable role in Si cycling. First, by exerting stress on plants, herbivores may induce Si uptake from the soil [143,144,151], hence increasing plant demand for soluble Si and thus the weathering of silicate minerals to meet this demand. Second, variations of herbivore feeding preferences and plant resistance to herbivory can alter plant community taxonomic and functional composition [429,430,431,432], as seen in several studies that show that grazing increases plant and community Si contents [150,151,154,156,160,433]. Therefore, herbivory may be a further catalyst of Si uptake from the soil and of ecosystem-level plant Si demand. Third, plant Si is also affected by ingestion within herbivores’ bodies due to chemical conditions—and possibly enzymatic activity—within herbivores’ digestive systems [255]. Like the case of dissolution in soils, and likely for the same reasons, phytolith morphotypes vary in dissolution rates [425,434]. Eventually, herbivores excrete these modified Si pools within feces [261], which by itself has chemical properties that affect soil chemistry. The fecal Si pool is more readily available for dissolution and, being in a more aqueous medium on top of the soil, is more susceptible to horizontal translocation and export from the ecosystem, making herbivores important players in Si export to rivers, lakes and oceans [21,25,255,258,382,383,384,385]. Finally, unlike plants, herbivores are not mobile but sessile and can, therefore, directly translocate Si horizontally following their diurnal movement. There are, for instance, several examples for accumulation of phytolith-rich deposits in livestock enclosures and following human secondary use of livestock feces [208,434,435,436,437,438,439,440,441].

In recent years, some scholars have advocated a coupling—or at the very least interaction—between the Si and carbon cycles, or more generally an effect of plant Si uptake and accumulation on the carbon cycle [1,21,303,333,405,417,422,442]. Several lines of evidence and reasoning—which we have discussed above—have led to this suggestion. One such line of evidence comes from the tradeoffs between plant Si content and some organic plant components, suggesting a Si–carbon tradeoff [1,7,207,302,303,304,305,306,307]. This is further augmented by evidence for plant Si uptake affecting plant stoichiometry and trading off with some nutrients [193,271,273,290]. These interactions and putative tradeoffs can act in various and even contrasting manners. On one hand, if Si can partially replace carbon, then its uptake can theoretically reduce carbon sequestration by plants. On the other hand, Si uptake and accumulation can improve carbon use efficiency and plant nutrition, and thus plant performance, hence having an overall positive effect on carbon sequestration by plants. Indeed, biomes dominated by Si-rich plants tend to be more productive [21]. Another path by which Si can improve ecosystem productivity is its involvement in soil appropriation. For example, soil ASi—of which a large part is of plant origin—improved soil water holding capacity and thus can reduce drought stress [103]. By taking up soluble Si from the soil and reincorporating it into the soil as ASi, plants imbalance soil Si forms and promote silicate mineral weathering [22,171,256,263,359,424], a CO_2_-consuming process [313,405,422,443]. Carbon occlusion within phytoliths—despite the controversies about the origin of this carbon [409,411,412,413,414,415]—is another mechanism by which plant Si can affect the carbon cycle [333,400,401,417,418,419], albeit only minor compared to other forms of plant carbon sequestration [1,400,420].

Silicon’s involvement in plant–herbivore interactions can also affect the carbon cycle. On one hand, herbivores accelerate Si cycling, which by extension may accelerate the carbon cycle, for example, by promoting silicate mineral dissolution. On the other hand, Si’s role in deterring herbivores or impairing their ability to ingest and digest plant biomass can reduce plant carbon turnover rates. The terrestrial Si cycle can also affect the marine Si and carbon cycles since accelerated Si cycling on land can enrich marine systems in Si and thus promote the growth of Si-rich biota (e.g., diatoms) and increase marine NPP [25,255]. Falkowski et al. [444], for example, suggested that the evolution of grasslands (dominated by grasses and grazers, two important Si-cyclers) released large amounts of Si into oceans and facilitated a surge of diatom evolution. Admittedly, some of the effects that Si uptake and cycling by plants has on the carbon cycle are uncertain or minor, but overall they may accumulate into an appreciable effect.

### 6.4. Silicon and Ecosystem Services

**Figure 5:**
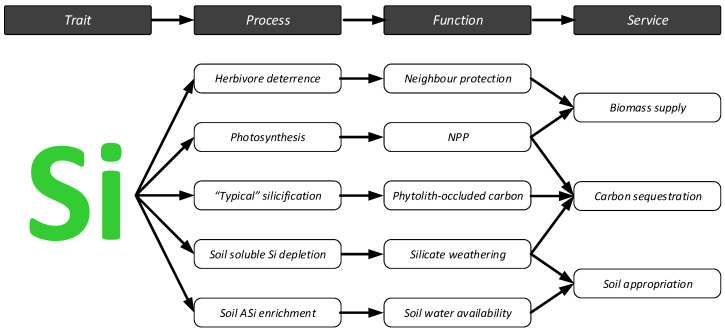
Several examples of how the trait (plant Si uptake and accumulation) drives processes that govern ecosystem functions and how these functions contribute to ecosystem services.

The multifunctionality of soil Si and its uptake and cycling by plants, as well as their many benefits for humans, imply that we should consider plant and herbivorous Si cyclers as providers of some ecosystem services (Figure 5). Many of these are supporting services, including soil appropriation and improvement of soil nutrient and water availability to plants [88], and possibly also increasing NPP [21], which is a provider of biomass (i.e., food for wildlife) and a carbon sink. Physically deterring herbivores and sometimes attracting their natural enemies can have secondary protective effects on neighboring plant species that lack these defenses [429,445,446]. Silicon cycling may even support evolution: its export to oceans may have contributed to diatom diversification [444], and its increased availability in soils may have contributed to the early diversification of some Si-rich angiosperm clades [23] (see comparable ideas regarding the evolutionary role of resource and nutrient cycling in general: [233,427,444,447,448]). Silicon can also provide some regulating services. For example, by promoting plant growth, carbon occlusion in phytoliths and silicate mineral weathering [400,405], as small as any one of these processes may be on its own [1,400,420]. Si uptake and accumulation is likely to have some contribution to removing atmospheric CO_2_ and thus regulating the atmospheric composition and global climate.

## 7. Conclusions

Many plants (including some prolific families like grasses) can take up Si from the soil, accumulate it within their tissues (where Si plays some biological roles) and then reincorporate it into the soil through litter creates an intricate network of feedback mechanisms in ecosystems. The bidirectional effects of soil properties on plant Si uptake and plant litter on soils suggests a true soil–plant continuum. To these, we should add the effects of Si in both soils and plants on ecosystems and global processes. These intricate feedback mechanisms in ecosystems make Si in the soil–plant continuum an important phenomenon in ecosystem functioning and a driver of some ecosystem services. These far exceed soil and plants, also affecting herbivores and the atmosphere. What we need now to push the field forward is detailed, interdisciplinary research with a focus on (i) the development of a standard protocol for the determination of bioavailable Si in soils, (ii) the understanding of the modes of action of Si in plants, i.e., an understanding of the pathways that increase plants’ resistance by Si accumulation, and (iii) gaining profound insights into the role of Si in influencing ecosystem structure and functioning. We consider our review as an urgent request and encouragement of future studies on these topics, which will help us to unravel the impact of Si on the soil–plant continuum, and thus on ecosystems.

## Figures and Tables

**Table 1:** Summary of main plant Si extraction and measurement techniques.

Extractant	Procedure	Reference
1% Na_2_CO_3_ solution	~30 mg plant material extracted in 1% Na_2_CO_3_ solution at 85 °C	[162,165]
0.5 M NaOH solution	~100 mg plant material extracted in 0.5 M NaOH solution at 85 °C	[162]
2-step HF	**Step 1**: ~100 mg plant material digested in a mixture of distilled water, nitric acid and hydrofluoric acid (40%) at 190 °C **Step 2**: hydrofluoric acid is neutralized by 10 mL a 4% boric acid solution at 150 °C	[166]
Lithium metaborate fusion	Plant material ashed at 500 °C. The ash is mixed with lithium meta-tetraborate at 1000 °C. The obtained bead is transferred into nitric acid.	[167,168,171]
Tiron (C_6_H_4_Na_2_O_8_S_2_)	Plant material added to tiron solution buffered at pH 10.5 at 85 °C.	[169]
No extractant, but XRF	~100 mg plant material homogenized to a powder, but calibration is required.	[170]
